# Impact of pretreatment characteristics and salvage strategy on outcome in patients with relapsed acute myeloid leukemia

**DOI:** 10.1038/leu.2017.22

**Published:** 2017-02-03

**Authors:** R F Schlenk, P Frech, D Weber, P Brossart, H-A Horst, D Kraemer, G Held, M Ringhoffer, A Burchardt, G Kobbe, K Götze, D Nachbaur, T Fischer, M Lübbert, H R Salih, H Salwender, G Wulf, E Koller, M Wattad, W Fiedler, S Kremers, H Kirchen, B Hertenstein, P Paschka, V I Gaidzik, V Teleanu, M Heuser, F Thol, K Döhner, J Krauter, A Ganser, H Döhner, and for the German-Austrian AMLSG

**Affiliations:** 1 Department of Internal Medicine III, University Hospital of Ulm, Ulm, Germany; 2 NCT Trial Center, National Center for Tumor Diseases (NCT), Heidelberg, Germany; 3 Department of Internal Medicine III, University Hospital of Bonn, Bonn, Germany; 4 Department of Internal Medicine II, University Hospital Schleswig-Holstein Campus Kiel, Kiel, Germany; 5 Department of Oncology and Hematology, Klinikum Oldenburg, Oldenburg, Germany; 6 Department of Internal Medicine I, University Hospital of Saarland, Homburg, Germany; 7 Department of Internal Medicine III, Städtisches Klinikum Karlsruhe, Karlsruhe, Germany; 8 Department of Hematology/Oncology, University-hospital Giessen, Giessen, Germany; 9 Department of Hematology, Oncology and Clinical Immunology, Heinrich-Heine-University Düsseldorf, Düsseldorf, Germany; 10 Department of Internal Medicine III, Technical University of Munich, Munich, Germany; 11 Department of Internal Medicine V, University Hospital Innsbruck, Innsbruck, Austria; 12 Department of Medicine III, Johannes Gutenberg-University Mainz, Mainz, Germany; 13 Department of Hematology and Oncology, University Hospital of Freiburg, Freiburg, Germany; 14 Department of Hematology and Oncology, Eberhard-Karls University, Tübingen, Germany; 15 Department of Hematology/Oncology, Asklepios Klinik Altona, Hamburg, Germany; 16 Department of Hematology and Oncology, University Hospital of Göttingen, Göttingen, Germany; 17 Department of Hematology/Oncology, Hanuschkrankenhaus, Wien, Austria; 18 Department of Hematology Oncology, Kliniken Essen-Süd, Essen, Germany; 19 Department of Internal Medicine II, University Medical Center Hamburg-Eppendorf, Hamburg, Germany; 20 Department of Hematology/Oncology, Caritas-Krankenhaus, Lebach, Germany; 21 Department of Hematology/Oncology, Krankenhaus der Barmherzigen Brüder, Trier, Germany; 22 Department of Internal Medicine I, Klinikum Bremen Mitte, Bremen, Germany; 23 Department of Hematology, Hemostasis, Oncology and Stem Cell Transplantation, Hannover Medical School, Hannover, Germany

Despite intensive induction and consolidation therapy in newly diagnosed patients with acute myeloid leukemia (AML), approximately half of the younger patients and about 80−90% of the older patients relapse^1, 2, 3^ and the majority of patients will succumb to their disease. The prognostic impact of clinical characteristics and genetic abnormalities, which are mostly assessed at initial diagnosis, is less clear. Currently, there is no commonly accepted standard for salvage treatment.^4^ Allogeneic hematopoietic cell transplantation (HCT) offers the highest chance of cure in this clinical circumstance.^2, 3, 4^ A study in 667 relapsed younger adults (15−60 years) of the HOVON study group revealed a longer relapse-free interval after first complete remission (CR1), presence of a core binding factor (CBF) AML at diagnosis, lower age at relapse and no previous stem-cell transplantation during first-line therapy as factors associated with more favorable prognosis.^5^ More recently, additional favorable genetic markers have been reported, such as biallelic *CEBPA* mutations^6^ and the genotype mutated *NPM1* in the absence of *FLT3*-ITD.^7^

In the current study, we evaluated pretreatment characteristics and type of salvage strategy in 1307 adult AML patients enrolled on five prospective first-line treatment trials of the German−Austrian AML Study Group (AMLSG) who experienced a relapse.

Between 1993 and 2009, 3324 adults were enrolled on five prospective treatment trials of the AMLSG for newly diagnosed AML (excluding acute promyelocytic leukemia): AMLHD93,^8^ AMLHD98A,^9^ AMLHD98B,^10^ AMLSG 06-04^11^ and AMLSG 07-04.^12^ These studies were approved by the institutional review boards of the participating centers. All patients gave informed consent to pretreatment cytogenetic and molecular genetic analyses as well as to treatment within the trials according to the Declaration of Helsinki. The present study included all patients treated in the above protocols^8, 9, 10, 11, 12^ who subsequently relapsed.

Response assessment followed the standard criteria.^3^ However, we report here on complete remission (CR) and CR with incomplete hematological recovery (CRi) as combined response end point, as full hematological recovery was frequently not achieved before the initiation of subsequent treatment (particularly allogeneic HCT). Overall survival was defined here as time between the date of relapse and death or last follow-up. At initial diagnosis chromosome banding analysis and molecular genetics were performed centrally in the AMLSG Laboratory for Cytogenetic and Molecular Diagnosis,^13^ and in a subset of patients sequencing data were available.^14^ Pairwise comparisons between patient subgroups were performed by the Mann−Whitney or Kruskal−Wallis test for continuous variables and by Fisher's exact test for categorical variables; multivariable logistic regression models were used to test the influence of covariates on response to salvage therapy. The Kaplan−Meier method was used to estimate the distribution of overall survival and an extended Cox model was used to evaluate prognostic variables. All statistical analyses were performed with the statistical software environment R, version 2.14.0, using the R packages rms, version 3.3-1, and cmprsk, version 2.2-2.^15^

Of the 3324 patients, 2170 (63%) achieved a CR1; a total of 1307 patients relapsed (*n*=953 after intensive chemotherapy, *n*=79 after autologous HCT and *n*=275 after allogeneic HCT). Median duration of CR1 was 274.5 days (range, 31 days−11.4 years). Of 1307 relapsed patients, 1120 patients (median age, 53.6 years; range, 18−82.1) received different salvage regimens and 187 patients (median age, 60.5 years; range, 25.0–85.3) received only palliative care including hydroxyurea. Median and 24-month survival of patients who received salvage therapy versus those who had palliative care were 7.89 months and 27.3% (95% confidence interval (CI), 24.8–30.2%), and 1.58 months and 3.7% (95% CI, 1.7–8.0%), respectively, underlining the very limited prognosis in patients experiencing AML relapse particularly in the absence of specific antineoplastic treatment.^1^ Salvage therapy included intensive chemotherapy (*n*=907, 81.0%), non-intensive chemotherapy including demethylating agents and low-dose cytarabine (*n*=62, 5.5%), direct allogeneic HCT (*n*=100, 8.9%), donor lymphocyte infusions (DLI) alone (*n*=17) or in combination with low-dose chemotherapy (*n*=13) in patients transplanted in CR1 (*n*=30, 2.7%) and investigational therapy (*n*=21 1.9%) (Table 1). Following salvage therapy, response (CR/CRi) was achieved overall in 430 of 1120 patients (38.4%) and according to applied treatment after intensive chemotherapy in 36.8%, after direct allogeneic HCT without prior salvage therapy in 73%, after non-intensive therapy in 11.3%, after investigational therapy in 19.0% and after DLI in 40%. In order to identify prognostic factors that may inform on CR/CRi achievement after intensive salvage therapy, we performed a logistic regression model with the end point CR/CRi after salvage therapy (*n*=907). This model revealed CR duration >18 months (odds ratio (OR), 1.58; *P*=0.01), biallelic *CEBPA* mutation (OR, 2.15; *P*=0.04) and CBF-AML (OR, 2.20; *P*<0.001) as favorable variables and adverse cytogenetics (OR, 0.58; *P*=0.02) and *FLT3*-ITD (OR, 0.56, *P*=0.003) independent of allelic ratio as unfavorable variables. Age, gender, type of AML, previous treatment in CR1 with autologous or allogeneic HCT, mutational status of *NPM1*, *DNMT3A*, *TET2*, *NRAS*, *KRAS*, *IDH1*, *IDH2*, *TP53*, *FLT3*-TKD and of genes coding for chromatin/spliceosome complex were not significantly correlated with achievement of a second CR/CRi. In contrast to already known favorable^5, 6^ and unfavorable^5^ genetic prognostic factors, our results indicate a very low probability for achieving a CR2 with standard intensive salvage therapy in patients exhibiting a *FLT3*-ITD, arguing in this clinical situation for experimental approaches (NCT02298166, NCT02039726, NCT02421939). Furthermore, mutated *NPM1* was not associated with response to salvage therapy, which is unexpected based on previous reports^7^ and very high CR1 rates.^1^ Based on the ORs in our model, we designed a score by summing up the respective factors of favorable (biallelic *CEBPA* mutation, +1; CBF-AML, +1; long CR-duration, +0.5) and unfavorable markers (adverse cytogenetics, −1; *FLT3*-ITD, −1), resulting in three groups with regard to response to salvage therapy: low CR/CRi probability (sum<0; *n*=281, CR-rate 25%), intermediate CR/CRi probability (sum=0; *n*=369, CR rate 36%) and high CR/CRi probability (sum>0; *n*=257, CR rate 54%). This simple score may be helpful in decision making, pro or against intensive salvage therapy in relapsed patients.

Allogeneic HCT was performed in 537 of 1120 (48%) patients receiving intensive therapy (48%); this includes 100 patients receiving direct allogeneic HCT without prior salvage therapy. The median time interval from diagnosis to allogeneic HCT was 78 days (range, 3−509 days) after relapse, with three patients receiving their transplant beyond 1 year after relapse. The donor types were as follows: *n*=146 matched related, *n*=366 matched unrelated and *n*=22 haplo-identical donors; *n*=3 were cord blood grafts. Allogeneic HCT was performed with refractory disease after salvage therapy in 247 patients, in CR/CRi after salvage therapy in 190 patients, and in 100 patients directly without salvage. Multivariable analysis on overall survival using an Andersen−Gill regression model taking into account the time dependency of allogeneic HCT on all intensively treated patients (*n*=1120) revealed CR1 duration <6 months (hazard ratio (HR), 1.46, *P*<0.001), allogeneic HCT during first-line therapy (HR, 1.26, *P*=0.03), age (HR for 10 years difference, 1.06, *P*=0.05), and *FLT3*-ITD (HR, 1.33, *P*=0.004) as unfavorable parameters, and CR1 duration >18 months (HR, 0.82, *P*=0.03), biallelic *CEBPA* mutation (HR, 0.54, *P*=0.004), CBF-AML (HR, 0.48, *P*<0.001) and an allogeneic HCT as treatment of relapse (HR, 0.65, *P*<0.0001) as favorable parameters for survival after relapse. Gender, type of AML, mutational status of *NPM1*, *DNMT3A*, *TET2*, *NRAS*, *K*-*RAS*, *IDH1*, *IDH2*, *TP53*, *FLT3*-TKD genes encoding the chromatin/spliceosome complex, and adverse cytogenetics were not significantly associated with survival. This extended Cox regression model including the time dependency of allogeneic HCT strongly supports that allogeneic HCT offers the highest chance of cure in this clinical situation.^2, 3^ Of note, there were 25 patients surviving longer than 3 years after relapse without proceeding to allogeneic HCT and 8 of them exhibited an inv(16)/t(16;16). These patients were in CR2 after intensive chemotherapy (*n*=7) or autologous HCT (*n*=1). In contrast, patients with short duration of CR1 (<6 months) and/or patients exhibiting a *FLT3*-ITD especially with a high allelic ratio have a dismal prognosis after relapse even after receiving an allogeneic HCT (*P*=0.027) and experimental approaches in addition to allogeneic HCT may be considered (NCT01468467, EudraCT 2010-018539-16). Furthermore, we analyzed the impact of our score on the end-point survival. This score revealed prognostic impact in the whole group (*P*<0.001) as well as in the treatment groups intensive salvage chemotherapy (*P*<0.001) and direct allogeneic HCT (*P*=0.03, Figure 1). Thus, in addition to being informative in terms of the probability of achieving a CR/CRi after intensive salvage therapy, our score was also prognostic with respect to survival after relapse. However, the score was prognostic, but not predictive in that nearly similar prognostic separation was seen between the three subgroups in patients receiving intensive salvage chemotherapy before tentatively proceeding towards an allogeneic HCT but also in those directly moving towards an allogeneic HCT (Figure 1).

In summary, the results of our study in relapsed AML indicate that response to salvage therapy is associated with specific genetic disease entities (CBF-AML, AML with biallelic *CEBPA* mutation), longer CR1 duration, absence of adverse cytogenetics and *FLT3*-ITD. A score integrating these factors into three groups is prognostic for the probability of achievement of a CR/CRi after intensive salvage chemotherapy and for survival after relapse in the whole group and in patients receiving direct allogeneic HCT without prior salvage therapy. *FLT3*-ITD appears to be an unfavorable prognostic marker in all analyses, underlining the need for targeted therapies in these patients.

## Figures and Tables

**Figure 1:**
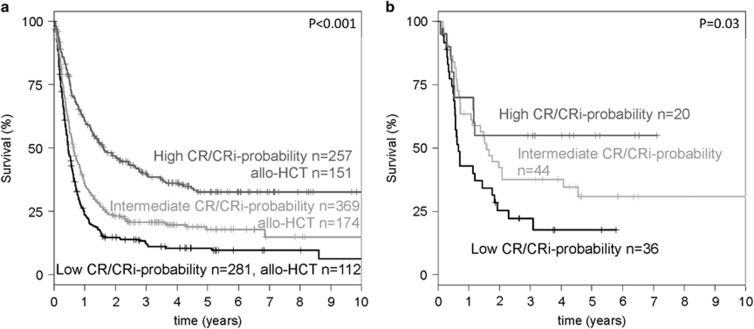
Kaplan−Meier plot illustrating the influence of the CR/CRi probability score on survival after relapse in patients receiving intensive salvage chemotherapy (**a**) or direct allogeneic HCT (**b**) as treatment of relapse. The score was based on the sum of favorable (+1) and unfavorable (−1) parameters defining three groups: low CR/CRi probability value<0; intermediate CR/CRi probability value=0, high CR/CRi probability value>0.

**Table 1:** Patient characteristics according to salvage therapy

	*Intensive (*n*=907)*	*Allogeneic HCT (*n*=100)*	*Non-intensive (*n*=62)*	*Experimental (*n*=21)*	*DLI (*n*=30)*	P*-value*
Age in years, median (range)	53.1 (17.1–80.8)	52.3 (18.3–73.5)	64.2 (28.0–82.1)	56.4 (39.9–77.9)	53.5 (18.8–73.7)	<0.0001
Missing	1	0	0	0	0	
						
Male gender, no. (%)	486 (53)	41 (41)	30 (48)	11 (52)	18 (60)	0.14
						
*WBC,*^a^ *10*^*9*^*/l*						
Median (range)	16 (0.4–394.4)	5.2 (0.5–294.9)	7 (0.6–177.5)	4.7 (0.7–190.1)	5.45 (0.2–150.8)	0.0005
Missing	12	1	1	1	0	
						
*Hemoglobin,*^a^ *g/dl*						
Median (range)	9.1 (2.5–16.5)	9.1 (3.1–13.4)	9.8 (5–14.7)	9 (5.9–12.1)	8.75 (3.5–14.1)	0.06
Missing	11	1	1	1	0	
						
*Platelets,*^a^ *10*^*9*^*/l*						
Median (range)	60 (4–916)	52 (6–449)	54 (12.7–418)	86 (14–574)	50.5 (4–529)	0.37
Missing	13	1	1	1	0	
						
*Bone marrow blasts,*^a^^,^^b^%						
Median (range)	80 (2–100)	70 (6–100)	80 (10–100)	46.5 (12–90)	80 (2–95)	0.05
Missing	73	7	3	3	1	
						
*Peripheral blood blasts,*^a^^,^^b^*%*						
Median (range)	44 (1–100)	33 (1−100)	34.5 (1–100)	25 (1–90)	28 (2–94)	0.19
Missing	150	19	10	6	6	
						
*Cytogenetics*						
CBF-AML, *n* (%)	115 (14.1)	5 (5.4)	3 (5.3)	0 (0)	1 (3.6)	0.005
Intermediate risk, *n* (%)	578 (70.8)	67 (72.8)	43 (75.4)	14 (77.8)	17 (60.7)	
Adverse risk,^c^ *n* (%)	124 (15.2)	20 (21.7)	11 (19.3)	4 (22.2)	10 (35.7)	
Missing	90	8	5	3	2	
						
*AML type*^a^						
* De novo* AML, *n* (%)	818 (90.5)	90 (90.0)	48 (78.7)	17 (81.0)	24 (80.0)	0.008
sAML, *n* (%)	33 (3.7)	6 (6.0)	6 (9.8)	3 (14.3)	4 (13.3)	
tAML, *n* (%)	53 (5.9)	4 (4.0)	7 (11.5)	1 (4.8)	2 (6.7)	
Missing	3	0	1	0	0	
						
*Mutated NPM1*^a^						
*n* (%)	230 (29.8)	18 (22.0)	15 (28.3)	5 (26.3)	4 (7.7)	0.07
Missing	136	18	9	2	3	
						
*Biallelic CEBPA mutation*^a^						
*n* (%)	34 (4.8)	3 (4.4)	3 (6.1)	0	1 (4.2)	0.96
Missing	199	32	13	3	6	
						
*FLT3-ITD*^a^						
*n* (%)	177 (22.2)	16 (18.6)	8 (15.1)	5 (26.3)	4 (14.8)	0.61
Missing	109	14	9	2	3	
						
*ELN risk*, n *(%)*^a^^,^^d^						
Favorable	242 (28.8)	14 (16.5)	12 (21.0)	0 (0)	2 (6.9)	0.002
Inter-1	224 (26.7)	30 (35.3)	18 (31.6)	9 (45)	9 (31.0)	
Inter-2	250 (29.7)	21 (24.7)	16 (28.1)	7(35)	8 (27.6)	
Adverse	124 (14.8)	20 (23.5)	11 (19.3)	4 (20)	10 (34.5)	

Abbreviations: AML, acute myeloid leukemia; CBF-AML, core-binding factor AML; DLI, donor lymphocyte infusion; ELN, European LeukemiaNet; FLT3, fms-related tyrosine kinase3; HCT, hematopoietic cell transplantation; ITD, internal tandem duplication; NPM1, nucleophosmin; sAML, AML after previous myelodysplastic syndrome; tAML, therapy-related AML; TKD, tyrosine kinase domain

WBC, white blood cells.

a At first diagnosis.

b In case of bone marrow blasts <20%, diagnosis of AML was established based on extramedullary disease or peripheral blood blasts >20%.

c According to ELN categorization.

d Updated ELN classification according to Döhner *et al.*^2^
